# Effects of Long Noncoding RNA HOTAIR Targeting miR-138 on Inflammatory Response and Oxidative Stress in Rat Cardiomyocytes Induced by Hypoxia and Reoxygenation

**DOI:** 10.1155/2021/4273274

**Published:** 2021-12-21

**Authors:** Guofeng Wang, Qi Wang, Weixue Xu

**Affiliations:** ^1^Department of Cardiology, The Fourth Affiliated Hospital of China Medical University, Shenyang, China; ^2^Department of General Surgery, Shengjing Hospital of China Medical University, Shenyang, China

## Abstract

**Objective:**

To investigate the effects of HOX transcript antisense RNA (HOTAIR) and miR-138 on inflammatory response and oxidative stress (OS) induced by IRI in rat cardiomyocytes.

**Methods:**

H9C2 cells were divided into the control group, H/R group, H/R+siRNA NC group, H/R+si-HOTAIR group, and H/R+si-HOTAIR+inhibitor group. Expression levels of HOTAIR, miR-138, and inflammatory factors were detected by quantitative reverse-transcription polymerase chain reaction (qRT-PCR). The double luciferase reporter gene assay was used to detect the targeting relationship between HOTAIR and miR-138.

**Results:**

Compared with the control group, the level of miR-138 and SOD in the H/R group was obviously reduced, while the expression levels of the HOTAIR, MDA, and NF-*κ*B pathway were obviously increased. Compared with the H/R group, the level of miR-138 and SOD in the H/R+si-HOTAIR group was obviously increased, and the expression levels of the HOTAIR, MDA, and NF-*κ*B pathway were obviously decreased. Compared with the H/R+si-HOTAIR group, the level of SOD in the H/R+si-HOTAIR+inhibitor group decreased; MDA content and the NF-*κ*B pathway expression level increased. In the double luciferase reporter gene assay, compared with the HOTAIR wt+NC group, the luciferase activity of the HOTAIR wt+miR-138 mimic group was obviously decreased.

**Conclusions:**

Silent HOTAIR can promote the expression of miR-138 and inhibit H/R-induced inflammatory response and OS by regulating the NF-*κ*B pathway, thus protecting cardiomyocytes.

## 1. Introduction

Acute myocardial infarction (AMI) is the acute manifestation of coronary heart disease and the main cause of death. Its basic pathological changes are the rupture of coronary artery plaques, thrombosis, significant reduction, or even interruption of blood supply, which eventually leads to severe ischemic and hypoxic lesions in some myocardial tissues [1]. If blood flow to the occluded vessels does not return, myocardial tissue in the infarct-related area will die. Timely reperfusion strategies, such as drug thrombolysis, percutaneous coronary intervention, and coronary artery bypass grafting, are conducive to the early opening of occluded blood vessels, effectively saving ischemic myocardial tissue, reducing infarction area, and improving cardiac function, and have greatly improved the prognosis of patients [2]. However, as the study of myocardial ischemia reperfusion proceeds further, the researchers found that although the reperfusion therapy can make the ischemia heart regain the blood perfusion in a short period of time to limit infarct area expansion, and many other benefits, it also leads to myocardial ischemia reperfusion treatment itself after more serious dysfunction and structural damage; this phenomenon is called myocardial ischemia reperfusion injury (MIRI) [3, 4]. Moreover, in 1960, Jennings et al. first proposed the MIRI hypothesis and named the description [5]. MIRI can cause the destruction of myocardial cell membrane and then change the myocardial ultrastructure; the scope of infarction is further expanded. Myocardial reversible or irreversible injury results in decreased systolic function of myocardium, and decreased ventricular threshold is accompanied by obvious refractory period shortening, which can be manifested as malignant arrhythmia, cardiac dysfunction, or even broken death [6]. In addition, MIRI can cause up to 50% of patients with successful PCI to have microcirculation disorder, which seriously affects the treatment effect and the prognosis of patients [7]. Therefore, how to reduce MIRI has become the focus and hot topic of research in the field of coronary heart disease.

The mechanism of the occurrence of MIRI has not been fully clarified. Currently, the mechanisms with more evidence include oxidative stress (OS), intracellular calcium overload, vascular endothelial injury, endoplasmic reticulum stress, and inflammatory response [8, 9]. Studies have shown that OS is one of the main causes leading to cardiac systolic dysfunction and myocardial reperfusion after ischemia; although the blood supply was restored, the increase in the number of generated oxygen free radicals (OFRs) in cells during ischemia and partly due to the overload of calcium ion will launch the apoptosis of myocardial cells, resulting in the structure and function of the myocardial cell injury [10]. Moreover, inflammatory reaction was also involved in the process of MIRI; the ischemia injury area has a variety of cytokines, including the interleukin family (IL-1, IL-6, IL-8, etc.), tumor necrosis factor (TNF-*α*), and NF-*κ*B; in addition to direct effect, inflammatory cytokines can also activate neutrophils and endothelial cells and cause OFRs, microcirculation thrombosis, and vascular endothelial cell dysfunction [11]. Therefore, the intervention of OS and inflammatory response is also an important way to reduce MIRI.

The results of the human genome research project confirmed that 2/3 of the gene sequences of more than 3 billion base pairs of humans have been transcribed, while less than 2% of nucleic acid sequences are used to encode proteins. The remaining 98% of DNA is mostly transcribed into RNA instead of being further translated into proteins. This type of RNA molecule is called the noncoding RNA (ncRNA) [12]. They regulate coding RNA and proteins at the transcriptional level and posttranscriptional level and play a complex and precise regulatory function in the process of biological evolution and disease occurrence and development. Among them, the long noncoding RNA (lncRNA) is a kind of ncRNA with a length greater than 200 nt, which is another endogenous RNA molecule after microRNA (miRNA) [13]. Many studies have shown that lncRNA plays an important role in the pathological process of I/R in many vital organs such as the heart, brain, liver, and kidney. In particular, some lncRNAs have been shown to be biomarkers in cardiac I/R studies, which provide potential therapeutic targets for the clinical prevention of MIRI. Through the establishment of the mouse cardiomyocyte model, the researchers found that lncRNA-MDRL could downregulate miRNA 361-miRNA 484 and reduce mitochondrial division and apoptosis, which ultimately protected MIRI [14]. lncRNA-HOTAIR was found to promote TNF-*α* protein expression in cardiomyocytes of LPS-induced sepsis mice by activating the NF-*κ*B pathway [15]. However, the role of lncRNA-HOTAIR in MIRI has not been reported. Through the NONCODE database (http://www.noncode.org), lncRNA HOTAIR was found to be widely conserved among species including humans and rats.

The nuclear transcription factor is a class of proteins, which has the function of binding to fixed nucleotide sequences in certain promoter regions to initiate gene transcription. NF-*κ*B is an important group of proteins, which can be seen in a variety of cells, such as vascular endothelial cells, vascular smooth muscle cells, and cardiomyocytes, and is involved in the gene regulation of a variety of physiological and pathological processes such as inflammation, immunity, cell proliferation, and apoptosis [16, 17].

In this study, rat myocardial cell line H9C2 was transfected into cells to silence lncRNA-HOTAIR, and then, anoxic reoxygenation was performed to simulate cardiac I/R for *in vitro* experiments to further investigate the role of lncRNA-HOTAIR in cardiac ischemia-reperfusion injury in rats.

## 2. Materials and Methods

### 2.1. Cell Culture

Rat myocardial cell line H9C2 (American Type Culture Collection, Manassas, VA, USA) was cultured in Dulbecco's modified Eagle medium (DMEM) (Life Technology, Gaithersburg, MD, USA) with 10% fetal bovine serum (FBS) (Life Technology, Gaithersburg, MD, USA) at 37°C, 5% CO_2_, and 95% humidity. Passage occurred when the cells were about 80% full.

### 2.2. Cell Transfection

H9C2 cells in the growing period were inoculated the day before transfection, and the cell density should reach 80%-90% coverage on the day of transfection. On the day of transfection, Lipofectamine 2000 transfection agents (Thermo Fisher Scientific, Waltham, MA, USA) were prepared into siRNA negative control and siRNA HOTAIR/Lipofectamine complexes, according to the instructions. The H/R+si-HOTAIR+inhibitor group was cotransfected with si-HOTAIR and miR-138 inhibitor according to the instructions of Lipofectamine 2000 transfection agents. Then, the mixture after incubation was directly added to the H9C2 cell medium. Finally, the cells were placed at 37°C in a 5% CO_2_ incubator for 4-6 h, and the fresh DMEM medium was replaced.

### 2.3. Establishment of Cell Hypoxia Reoxygenation Model

The cell hypoxic reoxygenation model was to simulate cell hypoxia and sugar-free environment *in vitro* to simulate ischemia and restore oxygen supply and saccharide environment to simulate reperfusion, so as to simulate the MIRI model. Specific steps are as follows: the cell culture medium of each group was replaced with serum-free low-glucose DMEM medium of 2 mL and put into an anoxic culture chamber full of 95% N_2_+5% CO_2_ mixed gas (oxygen concentration less than 1%). The anoxic culture medium was incubated at 37°C for 1 h to simulate ischemia. During the reperfusion period, the cell culture plate was taken from the anoxic culture box, and the culture medium was replaced with the normal DMEM medium containing serum and sugar of 2 mL. The mixed gas of 95% N_2_+5% CO_2_ was injected into the air inlet with the flow rate of 5 L/min and reoxygenated at 37°C for 3 h.

### 2.4. Western Blot Test

Cells of each group were collected, with each group containing about 4 × 10^6^ cells, and lysed in 200 *μ*L lysate containing protease inhibitor, lysed at 4°C for 40 min, and centrifuged at 15,000 RPM for 20 min; then, the supernatant was taken, and protein was quantified by the bicinchoninic acid (BCA) kit (Jian Cheng, Nanjing, China). After mixing with a 4x sample buffer, the sample was boiled for 5 min. Then, the 40 *μ*g sample was electrophoresed in 8-10% sodium dodecyl sulphate (SDS) polyacrylamide gel for 2 h and then transferred to a polyvinylidene difluoride (PVDF, Thermo Fisher Scientific, Waltham, MA, USA) membrane. After being sealed with 5% skim milk for 2 h, the membrane was cut out according to the molecular weight, and primary antibodies (IL-1*β*, Abcam, Cambridge, MA, USA, Rabbit, 1 : 1000; IL-6, Abcam, Cambridge, MA, USA, Rabbit, 1 : 3000; TNF-*α*, Abcam, Cambridge, MA, USA, Rabbit, 1 : 3000; NF-*κ*B p65, Abcam, Cambridge, MA, USA, Mouse, 1 : 2000; IKK, Abcam, Cambridge, MA, USA, Mouse, 1 : 2000; p-IKB*α*, Abcam, Cambridge, MA, USA, Rabbit, 1 : 3000; GAPDH, Proteintech, Rosemont, IL, USA, 1 : 5000) were added at 4°C overnight. After washing with phosphate-buffered saline (PBS) for 3 times, anti-rabbit or mouse IgG secondary antibody (1 : 1 000 dilution, Yifei Xue Biotechnology, Nanjing, China) was added for 1 h at room temperature. We used enhanced chemiluminescence (ECL) technology (Jian Cheng, Nanjing, China) to expose the target protein, and the image analysis system was used for photography and analysis.

### 2.5. Total RNA Extraction and Quantitative Reverse-Transcription Polymerase Chain Reaction (qRT-PCR)

After the cells in each group were collected, a TRIzol reagent (Thermo Fisher Scientific, Waltham, MA, USA) was used to extract RNA from each group, complementary deoxyribose nucleic acid (cDNA) was synthesized according to the reverse transcription kit (Thermo Fisher Scientific, Waltham, MA, USA), and then, PCR reaction was performed with a PCR apparatus (Becton Dickinson, Heidelberg, Germany). The cyclic conditions were as follows: predenaturation at 95°C for 10 min, denaturation at 95°C for 15 s, and annealing at 63°C for 1 min, a total of 40 cycles. The mRNA levels of each group were detected by SsoFast EvaGreen Supermix kit (Yi Hui, Shanghai, China), and the results were calculated by the 2^-∆∆CT^ method. The primers used are shown in Table 1.

### 2.6. Enzyme-Linked Immunosorbent Assay (ELISA)

After grouping treatment according to the test methods described above, the supernatant of cells in each group was collected, and the contents of MDA, and the activities of SOD and inflammation-related factors (TNF-*α*, IL-1*β*, IL-6) were detected according to the instructions of the kit (Jian Cheng, Nanjing, China).

### 2.7. Luciferase Test

Bioinformatics analysis and prediction showed that the possible binding site of miR-138 was located in the lncRNA HOTAIR 2142~2163 bp region. The HOTAIR Lenti-reporter-Luciferase wild-type vector and its mutant vector were constructed separately (Thermo Fisher Scientific, Waltham, MA, USA). The cells were divided into four groups: HOTAIR wt-NC, HOTAIR mut-NC, HOTAIR wt+miR-138, and HOTAIR mut+miR-138 mimics. Cells were plated in 24-well plates and incubated for 24 h. The next day, the wild-type vector (200 ng) and pRL-CMV vector (20 ng) were transfected into the HOTAIR wt+NC and HOTAIR wt+miR-138 mimic groups; the mutant vector (200 ng) and the Prl-CMV vector (20 ng) were transfected into HOTAIR mut-NC and HOTAIR mut+miR 138 mimic groups. At the same time, miR-138 mimics was transfected in the HOTAIR wt+miR-138 mimic and HOTAIR mut+miR-138 mimic groups. 48 h after transfection, luciferase activity was detected using a luciferase reporter detection system (Thermo Fisher Scientific, Waltham, MA, USA).

### 2.8. Immunofluorescence

After the cells were fixed with 4% paraformaldehyde for 15 minutes, they were washed with 0.01 mol/L PBS buffer solution for 3 times and then sealed with 10% goat serum + 0.03% Triton X-100 blocking solution (Elabscience, Wuhan, China) at room temperature for 2 h, and then, a primary antibody (SOD1, Abcam, Cambridge, MA, USA, Rabbit, 1 : 3000) was added, placed in a refrigerator at 4°C overnight, and then washed with 0.01 mol/L PBS buffer solution. The sheep anti-mouse secondary antibody (Jian Cheng, Nanjing, China) was added, incubated at room temperature for 2 h, and then washed with 0.01 mol/L PBS buffer solution. The cell nucleus was stained by 4′,6-diamidino-2-phenylindole (DAPI) (Jian Cheng, Nanjing, China) in the dark, and antifade solution (Jian Cheng, Nanjing, China) was added to the film and stored at 4°C for observation under fluorescence microscope (Keyence, Shanghai, China).

### 2.9. Statistical Analysis

Data analysis was performed using Statistical Product and Service Solutions (SPSS) 21.0 software (IBM, Armonk, NY, USA). Measurement data were expressed as mean ± standard deviation. Differences between two groups were analyzed by using Student's *t*-test. Comparison between multiple groups was done using one-way ANOVA test followed by Post Hoc Test (Least Significant Difference). Chi-square test was used for enumeration data. *P* < 0.05 was considered statistically significant.

## 3. Results

### 3.1. Effect of H/R on H9C2 Cells

**Figure 1:**
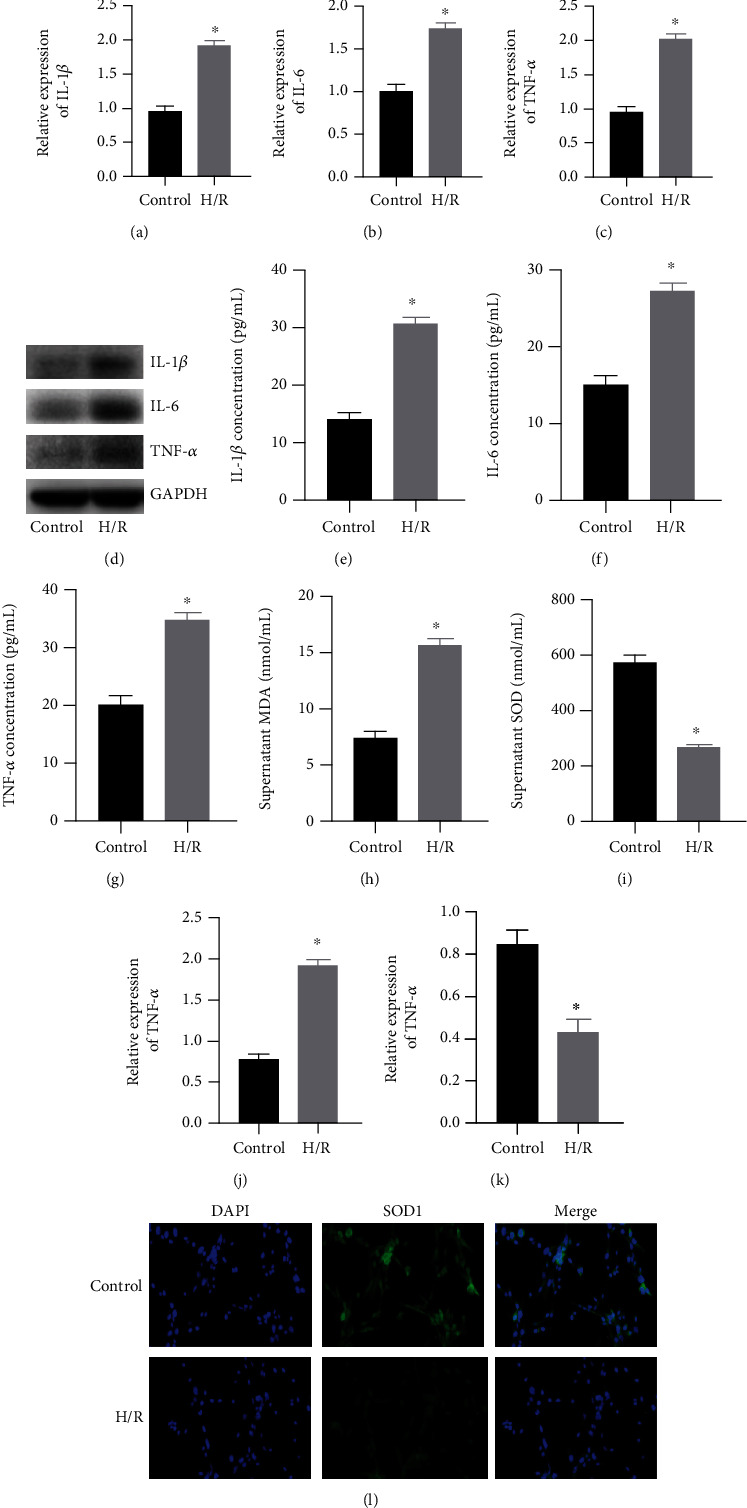
Effect of H/R on H9C2 cells. (a) qRT-PCR was used to detect the expression of IL-1*β*. (b) qRT-PCR was used to detect the expression of IL-6. (c) qRT-PCR was used to detect the expression of TNF-*α*. (d) WB was used to detect the expression of IL-1*β*, IL-6, and TNF-*α*. (e) ELISA was used to detect the content of IL-1*β*. (f) ELISA was used to detect the content of IL-6. (g) ELISA was used to detect the content of TNF-*α*. (h) ELISA was used to detect the content of MDA. (i) ELISA was used to detect the activity of SOD. (j) qRT-PCR was used to detect the expression of HOTAIR. (k) qRT-PCR was used to detect the expression of miR-138. (l) Immunofluorescence staining was used to detect the expression of SOD1 (magnification: 400x). (“∗” indicates statistical difference from the control group, *P* < 0.05).

Compared with the control group, H9C2 cells in the H/R group had an obvious inflammatory response. IL-1*β*, IL-6, and TNF-*α* were detected by qRT-PCR (Figures 1(a)–1(c)). H/R induced an inflammatory response and promoted the increased expression of IL-*β*, IL-6, and TNF-*α*. At the same time, WB (Figure 1(d)) and ELISA (Figures 1(e)–1(g)) results confirm this. Next, we used the kit to detect the MDA content and SOD activity in the cell supernatant (Figures 1(h) and 1(i)) and found that H/R caused a redox imbalance, resulting in a decrease in SOD activity and an increase in MDA content. At the same time, we tested the expression of SOD1 in each group (Figure 1(j)). The results of immunofluorescence staining showed that the expression of SOD1 in the H/R group was obviously reduced. Subsequently, qRT-PCR detection revealed that the expression of lncRNA-HOTAIR increased (Figure 1(k)), while the expression of miR-138 (Figure 1(l)) decreased in the H/R group.

### 3.2. Effects of Silenced lncRNA-HOTAIR on H9C2 Cells

**Figure 2:**
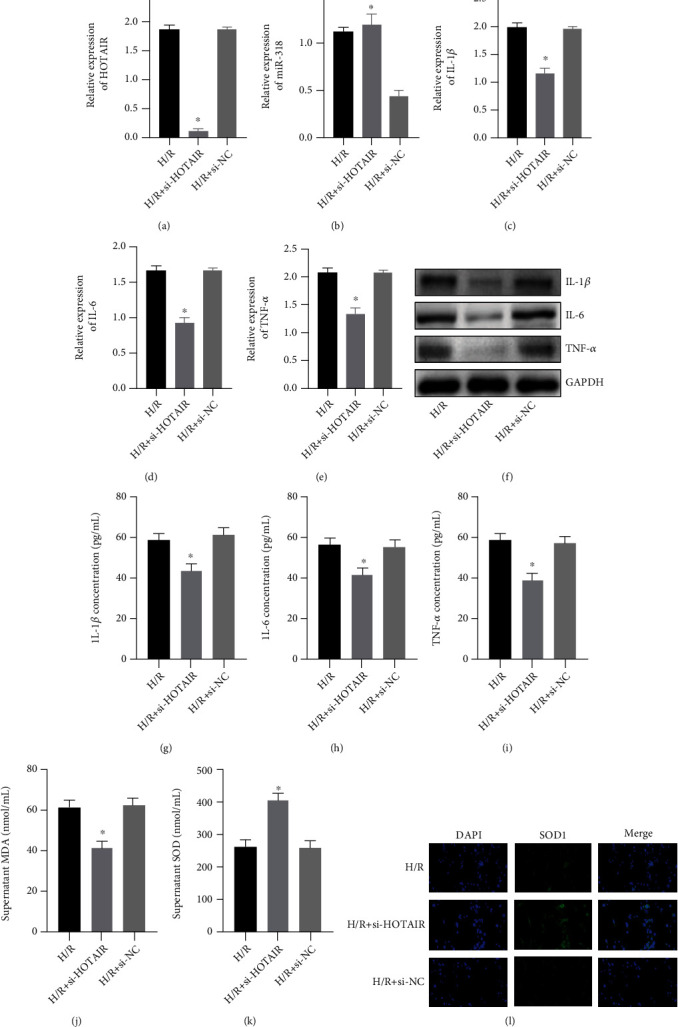
Effects of silenced lncRNA-HOTAIR on H9C2 cells. (a) qRT-PCR was used to detect the expression of HOTAIR. (b) qRT-PCR was used to detect the expression of miR-138. (c) qRT-PCR was used to detect the expression of IL-1*β*. (d) qRT-PCR was used to detect the expression of IL-6. (e) qRT-PCR was used to detect the expression of TNF-*α*. (f) WB was used to detect the expression of IL-1*β*, IL-6, and TNF-*α*. (g) ELISA was used to detect the content of IL-1*β*. (h) ELISA was used to detect the content of IL-6. (i) ELISA was used to detect the content of TNF-*α*. (j) ELISA was used to detect the content of MDA. (k) ELISA was used to detect the activity of SOD. (l) Immunofluorescence staining was used to detect the expression of SOD1 (magnification: 400x). (“∗” indicates statistical difference from the H/R group, *P* < 0.05).

Based on the above qRT-PCR test results, we established a transient transfected cell model of silent lncRNA-HOTAIR (si-HOTAIR) and then treated the cells with hypoxia and reoxygenation. First, we detected by qRT-PCR that HOTAIR expression in the si-HOTAIR+H/R group was obviously reduced (Figure 2(a)). At the same time, we also tested the miR-138 expression and found that the miR-138 expression was increased in the si-HOTAIR+H/R group (Figure 2(b)). Subsequently, the expression of IL-1*β*, IL-6, and TNF-*α* was detected by qRT-PCR (Figures 2(c)–2(e)). It was found that the expression of inflammatory factors was obviously suppressed in the si-HOTAIR+H/R group. WB (Figure 2(f)) and ELISA (Figures 2(g)–2(i)) also found that silencing lncRNA-HOTAIR can reduce the H/R-induced inflammation. Next, we measured the MDA content (Figure 2(j)) and the SOD activity (Figure 2(k)) in the cell supernatant and found that silencing lncRNA-HOTAIR can alleviate the redox imbalance caused by H/R, increase the SOD activity, and inhibit the increase in MDA content. At the same time, we detected SOD1 expression by immunofluorescence and found that SOD1 expression was low in the H/R group and the H/R+siRNA NC group, while SOD1 expression was relatively high in the H/R+si-HOTAIR group (Figure 2(l)).

### 3.3. Effect of Silenced miR-138 on H9C2 Cells

**Figure 3:**
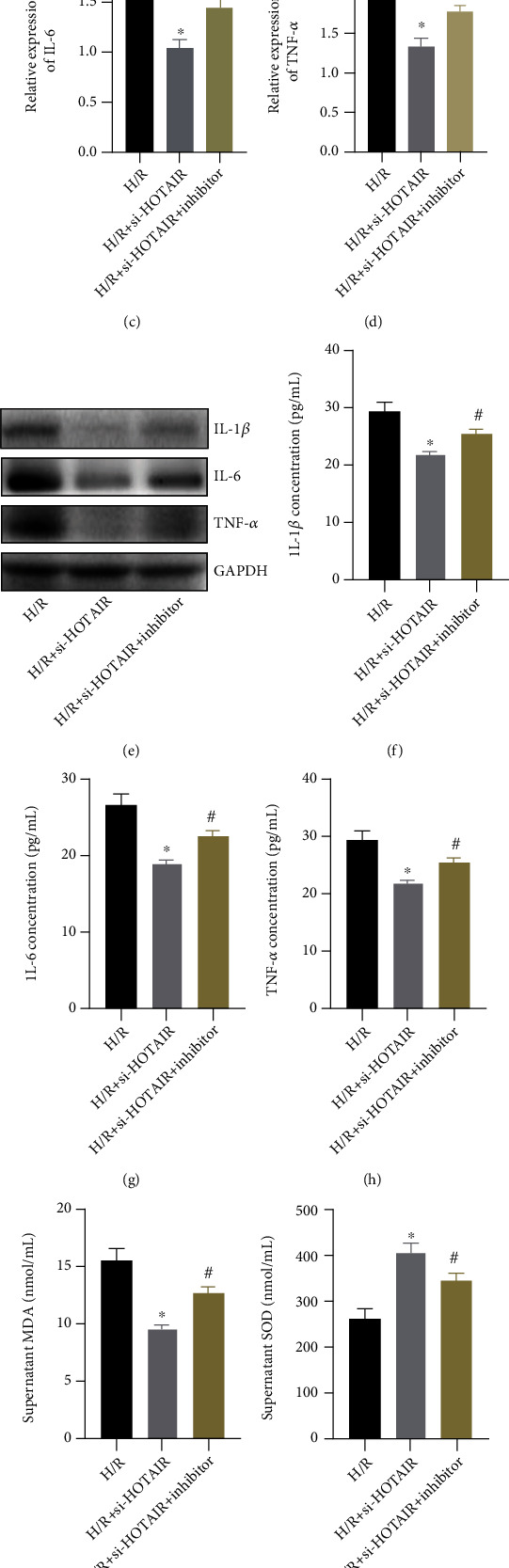
Effect of silenced miR-138 on H9C2 cells. (a) The luciferase reporter assay was used to detect the targeting relationship between miR-138 and HOTAIR. (“∗” indicates statistical difference from the HOTAIR wt+miR-138 NC group *P* < 0.05.) (b) qRT-PCR was used to detect the expression of IL-1*β*. (c) qRT-PCR was used to detect the expression of IL-6. (d) qRT-PCR was used to detect the expression of TNF-*α*. (e) WB was used to detect the expression of IL-1*β*, IL-6, and TNF-*α*. (f) ELISA was used to detect the content of IL-1*β*. (g) ELISA was used to detect the content of IL-6. (h) ELISA was used to detect the content of TNF-*α*. (i) ELISA was used to detect the content of MDA. (j) ELISA was used to detect the activity of SOD. (k) Immunofluorescence staining was used to detect the expression of SOD1 (magnification: 400x). (“∗” indicates statistical difference from the H/R group, *P* < 0.05, and “#” indicates statistical difference from the H/R+si-HOTAIR group, *P* < 0.05).

Based on the above results and literature review, it was confirmed that miR-138 has a targeting relationship with lncRNA-HOTAIR. At the same time, the luciferase test found that compared with the HOTAIR wt+NC group, the luciferase activity of the HOTAIR wt+miR-138 mimic group was obviously reduced, and the difference was statistically significant (Figure 3(a)). There was no significant difference in luciferase activity between the HOTAIR mut+NC group and the HOTAIR mut+miR-138 mimic group. It was verified that miR-138 can target HOTAIR. So, does silencing miR-138 inhibit the effect of lncRNA-HOTAIR on H/R-induced OS and inflammation? Therefore, we divided H9C2 cells into 3 groups: H/R, H/R+si-HOTAIR, and H/R+si-HOTAIR+inhibitor. Then, the expression of IL-1*β*, IL-6, and TNF-*α* was detected by qRT-PCR (Figures 3(b)–3(d)), WB (Figure 3(e)), and ELISA (Figures 3(f)–3(h)). It was found that the degree of inflammation in the H/R+si-HOTAIR+inhibitor group was obviously higher than that in the H/R+si-HOTAIR group. Detection of MDA content (Figure 3(i)) and SOD activity (Figure 3(j)) in cell supernatants revealed that miR-138 inhibitor can partially inhibit the effect of si-HOTAIR, resulting in a decrease in SOD activity and an increase in MDA content. Immunofluorescence staining for SOD1 revealed that SOD1 expression was lower in the H/R+si-HOTAIR+inhibitor group than in the H/R+si-HOTAIR group (Figure 3(k)).

### 3.4. Silencing lncRNA-HOTAIR Inhibits NF-*κ*B Pathway Activation in H9C2 Cells

**Figure 4:**
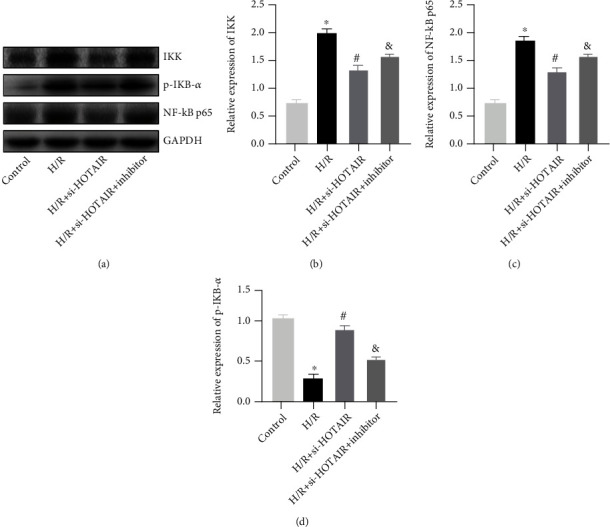
Silencing lncRNA-HOTAIR inhibits NF-KB pathway activation in H9C2 cells. (a) WB was used to detect the expression of IKK, p-IKB-*α*, and NF-*κ*B p65. (b) qRT-PCR was used to detect the expression of IKK. (c) qRT-PCR was used to detect the expression of NF-*κ*B p65. (d) qRT-PCR was used to detect the expression of IKB-*α*. (“∗” indicates statistical difference from the control group, *P* < 0.05; “#” indicates statistical difference from the H/R group, *P* < 0.05; and “&” indicates statistical difference from the H/R+si-HOTAIR group, *P* < 0.05).

Compared with the control group, the protein expression levels of IKK, p-IKB*α*, and NF-*κ*B p65 in the H/R group increased obviously. Compared with the H/R group, the expression levels of IKK, p-IKB*α*, and NF-*κ*B p65 in the H/R+si-HOTAIR group were obviously reduced. Conversely, compared with the H/R+si-HOTAIR group, the expression levels of IKK, p-IKB*α*, and NF-*κ*B p65 in the H/R+si-HOTAIR+inhibitor group were obviously increased (Figure 4(a)). At the same time, the qRT-PCR detection, IKK, and NF-*κ*B p65 expression were similar to WB results, and IKB-*α* expression was lowest in the H/R group, and the expression was increased in the H/R+si-HOTAIR group, while the inhibitor can inhibit some of the effects of si-HOTAIR (Figures 4(b)–4(d)).

## 4. Discussion

Previous literature has reported that HOTAIR can activate the NF-*κ*B signaling pathway and activate the myocardial inflammatory response by promoting phosphorylation of the NF-*κ*B p65 subunit [18]. miR-138 can reduce myocardial cell apoptosis caused by hypoxia through the MLK3/JNK/c-Jun signaling pathway [19]. In addition, studies have confirmed that HOTAIR and miR-138 can interact with each other to affect LPS-induced rheumatoid arthritis *in vivo* [18]. This study was the first to explore the mechanism of the interaction between miR-138 and HOTAIR in the myocardial inflammation induced by H/R. In this study, it was found that the expression level of HOTAIR in H9C2 cells after H/R treatment was obviously increased, and the expression level of miR-138 was obviously decreased. The expression level of miR-138 was obviously increased after the silence of HOTAIR expression, indicating that HOTAIR and miR-138 did interact. Through luciferase reporter gene detection, it was found that HOTAIR could directly act on miR-138, which confirmed previous studies.

Inflammatory cytokines are involved in the MIRI process. Studies have shown that TNF-*α* protein and IL-1*β* protein jointly initiate the inflammatory response and have a negative inotropic effect on the myocardium, while the negative regulatory effect of IL-6 on the myocardial contractility is related to each other through the protein kinase pathway [20]. In this study, it was found that the silencing of HOTAIR could inhibit the expression of inflammatory cytokines TNF-*α*, IL-1*β*, and IL-6 in the H/R model, indicating that HOTAIR was involved in the expression of inflammatory cytokines. OS also plays a key role in MIRI, which induces excessive production of reactive oxygen species (ROS) in mitochondria, leading to an oxidation/antioxidant imbalance. ROS damage the myocardial cell membrane, leading to the production of a large amount of lipid peroxide MDA and the depletion of SOD activity. In this study, it was found that by silencing the expression of HOTAIR, the activity of SOD was restored and the content of MDA decreased, indicating that HOTAIR was involved in the OS of H9C2 cells. Further silencing of miR-138 revealed that the activity of SOD was decreased and the content of MDA was increased, indicating that miR-138 had an inhibitory effect on H/R-induced OS in H9C2 cells.

The NF-*κ*B signaling pathway is widely involved in various inflammatory responses. In general, NF-*κ*B family proteins exist in the form of isomeric p50-p65 and bind to inhibitor kappaB (IKB) in an inactivated state, which is located in the cytoplasm. In the presence of inducers such as TNF-*α*, IL-1, and IL-6, the IKB kinase is activated, phosphorylates IKB, causes it to release p50-p65, and is eventually degraded by proteasomes. At this time, p50-p65 exposed nuclear localization signal, transport into the nucleus, as a transcription factor to bind to various target DNA sequences [21, 22]. In order to explore whether the NF-*κ*B signaling pathway was regulated by HOTAIR and miR-138 in H9C2 cells during the H/R process, in this study, the expression levels of IKK, p-IKB*α*, and NF-*κ*B p65 were detected *in vitro*. It was found that the expressions of IKK, p-IKB*α*, and NF-*κ*B p65 were obviously inhibited by the silence of HOTAIR, indicating that HOTAIR was involved in the activation of NF-*κ*B and promotes the inflammatory response. At the same time, we further silenced miR-138 with inhibitors and found that the expression levels of IKK, p-IKB*α*, and NF-*κ*B p65 were obviously higher than those in the H/R+si-HOTAIR group, indicating that miR-138 could inhibit the activation of NF-*κ*B and thus reduce the inflammatory response.

In summary, lncRNA HOTAIR was involved in the expression of inflammatory factors in cardiomyocytes induced by H/R in H9C2 cells and the occurrence of OS and has an activation effect on NF-*κ*B, while miR-138 can inhibit OS and the expression of inflammatory factors by inhibiting the NF-*κ*B signaling pathway. HOTAIR can interact with miR-138 in H9C2 cells, resulting in miR-138 silencing. Therefore, HOTAIR can regulate the inflammatory response and OS of H9C2 cells by silencing miR-138. Furthermore, in combination with the previously reported literature, we speculated that HOTAIR and miR-138 play a regulatory role in different pathways of myocardial inflammatory response induced by H/R.

## 5. Conclusions

Silent HOTAIR can promote the expression of miR-138 and inhibit H/R-induced inflammatory response and OS by regulating the NF-KB pathway, thus protecting cardiomyocytes, which provides a new therapeutic approach for the maintenance of heart function in the treatment of MIRI.

## Figures and Tables

**Table 1:** RT-PCR primers.

Gene name	Forward (5′ > 3′)	Reverse (5′ > 3′)
IL-1*β*	GCAACTGTTCCTGAACTCAACT	ATCTTTTGGGGTCCGTCAACT
IL-6	TAGTCCTTCCTACCCCAATTTCC	TTGGTCCTTAGCCACTCCTTC
TNF-*α*	CCTCTCTCTAATCAGCCCTCTG	GAGGACCTGGGAGTAGATGAG
IKK	GTCAGGACCGTGTTCTCAAGG	GCTTCTTTGATGTTACTGAGGGC
IKB-*α*	GGATCTAGCAGCTACGTACG	TTAGGACCTGACGTAACACG
NF-*κ*B p65	ACTGCCGGGATGGCTACTAT	TCTGGATTCGCTGGCTAATGG
GAPDH	ACAACTTTGGTATCGTGGAAGG	GCCATCACGCCACAGTTTC

RT-PCR: quantitative reverse-transcription polymerase chain reaction.

## Data Availability

The datasets used and analyzed during the current study are available from the corresponding author on reasonable request.
